# The efficacy and safety of acupuncture-related therapy in the treatment of rheumatoid arthritis: A protocol for systematic review and network meta-analysis

**DOI:** 10.1097/MD.0000000000026859

**Published:** 2021-08-13

**Authors:** Jingwen Shang, Jia Xu, Zilong Zhang, LinLing Tian, Yongyang He

**Affiliations:** a Chengdu Eighth People's Hospital (Geriatric Hospital of Chengdu Medical College), Chengdu, Sichuan Province, China; b Second Provincial People's Hospital of Gansu, Gansu Province, China; c Chengdu University of TCM, Chengdu, China.

**Keywords:** acupuncture-related therapy, network meta-analysis, protocol, rheumatoid arthritis

## Abstract

**Background:**

Rheumatoid arthritis (RA) has seriously affected the quality of life of patients with its refractory, recurrent, and disabled characteristics, and has become a major public health problem. Previous studies have confirmed that acupuncture and moxibustion have a reliable effect on RA, but there are many forms of acupuncture and moxibustion, and the efficacy of each form is different. This study is to evaluate the clinical efficacy of different acupuncture-related therapies in the treatment of RA by means of network meta-analysis.

**Methods:**

According to the retrieval strategy, we retrieved the randomized controlled studies on acupuncture-related therapy for RA from China National Knowledge Infrastructure, Wanfang, VIP, China Biomedicine, PubMed, Embase, Web of Science, and The Cochrane Library databases from the establishment of the database to July 2021. We assessed the quality of the studies using the Cochrane Risk Bias Assessment Tool and assessed the strength of the evidence using the Grading of Recommendation Assessment, Development, and Evaluation methodology. All data analyses were performed by Revman5.3, Gemtc 0.14.3, and Stata 14.0.

**Results:**

This study is to evaluate the efficacy of different acupuncture-related therapies in the treatment of RA by evaluating the total effective rate, pain scores, joint function scores, quality of life scores, laboratory indicators, adverse reactions, etc.

**Conclusion:**

This study will provide a reliable evidence-based basis for the selection of the best acupuncture form for the treatment of RA.

**Ethics and dissemination:**

Private information from individuals will not be published. This systematic review also does not involve endangering participant rights. Ethical approval will not be required. The results may be published in a peer-reviewed journal or disseminated at relevant conferences.

## 1 Introduction

Rheumatoid arthritis (RA) is an autoimmune disease^[1,2]^ featuring chronic and erosive polyarthritis, which is pathologically characterized by inflammatory cell infiltration, joint synovial hyperplasia, associated with progressive damage to articular cartilage and subcartilage bone, eventually leading to joint dysfunction and even deformed.^[3,4]^ RA can occur at any age, the peak of which is between 30 and 60 years old, and the global prevalence is up to 0.5% to 1%.^[1]^ With a long course of the disease and low cure rate, RA can lead to joint deformity, dysfunction, and even premature death,^[5]^ which not only leads to a decline in the quality of life of patients but also brings a heavy burden on society and economy.^[6]^ Until now, RA remains a formidable clinical challenge.

At present, there is no clinical radical cure for RA, and the disease is mainly modified by non-steroidal anti-inflammatory drugs, conventional disease-modifying antirheumatic drugs, glucocorticoids, biological disease-modifying antirheumatic drugs, and other drugs are used to control and delay the progression of patients’ disease and improve the quality of life of patients.^[7]^ Although drug therapy can alleviate RA symptoms and inflammation to a large extent, it is difficult for patients to tolerate due to the long period of traditional drug therapy and large adverse reactions.^[8]^ Therefore, RA patients still need complementary and alternative therapies to seek additional remission or therapies with fewer side effects. About 30% to 60% of patients are using some form of complementary alternative.^[9]^

Acupuncture therapy, as a complementary and alternative therapy, has definite efficacy in the treatment of RA, mainly showing obvious advantages in improving patients’ clinical symptoms, delaying disease progression, alleviating pain, etc^[10]^ with small adverse reactions and low cost. Acupuncture takes many forms and has different effects, and previous studies have shown that conventional acupuncture improves joint function and quality of life in RA patients compared to western medicines.^[11]^ Bee acupuncture therapy is obviously superior in terms of total effective rate, erythrocyte sedimentation rate, C reactive protein, etc than western medicine.^[12]^ Electroacupuncture can effectively reduce the level of blood biochemical indicators, relieve the symptoms of patients’ joints, and the efficacy is better than western medicine.^[13]^ Current evidence shows that different forms of acupuncture therapies are effective on RA, but a direct comparison of the efficacy between different acupuncture therapies is still lacking. Meta-analysis is 1 of the highest levels of evidence in evidence-based research. However, traditional pairwise meta-analysis is difficult to compare the efficacy of multiple drugs simultaneously. Network meta-analysis (NMA) is a further development based on a traditional pairwise meta-analysis.^[14]^ According to the current clinical research data, NMA can simultaneously complete the direct and indirect comparison between different acupuncture therapies, and further comprehensive analysis of the direct and indirect comparison results, to obtain the efficacy of different acupuncture therapies ranking. Therefore, this study uses the NMA method to compare the efficacy of different acupuncture therapies on patients with RA, so as to provide evidence for the selection of the optimal acupuncture treatment program in the clinical treatment of RA.

## 2 Methods

### 2.1 Protocol register

This NMA was conducted according to the Preferred Reporting Items for Systematic Reviews and Meta-Analyses for NMA guidelines.^[15]^ Moreover, it has been registered on open science framework (Registration number: DOI 10.17605/OSF.IO/FRDXA).

### 2.2 Ethics

Since the protocol does not require patient recruitment and the collection of personal information, it does not require approval from Ethics Committee.

### 2.3 Eligibility criteria

(1) Study type: randomized controlled trials (RCTs), not limited to blind methods, language limited to Chinese and English.
(2) Study object: patients with RA diagnosed according to clear diagnostic criteria (the diagnostic labeling refers to the RA diagnostic criteria^[16]^ established by the 2010 American College of Rheumatology and the European League Against Rheumatism), gender and age were not limited.
(3) Interventions: in the treatment group, conventional acupuncture, warm needle, electroacupuncture, fire needle, moxibustion, auricular acupuncture, auricular acupoint sticking, acupoint embedding, acupoint injection, bee acupuncture were used; the control group was treated with western medicine, sham acupuncture, and sham moxibustion; or a comparison between different types of acupuncture.
(4) Exclusion criteria
  i. Studies of which the subjects did not meet the inclusion criteria, such as patients with other arthritis.
  ii. Studies in which the acupuncture combined therapy, such as acupuncture combined with moxibustion, acupuncture combined with auricular acupuncture treatment, were used.
  iii. Studies in which other TCM treatments, such as cupping and TCM compound, were applied in the 2 groups.
  iv. For repeated publications, select the literature with the most complete data.
  v. The type of publication is abstract, or there is no specific data of relevant indicators in the article, but the relevant data cannot be obtained by contacting the author of the article.

### 2.4 Outcome indicators

(1) Main outcome indicators: total effective rate, pain scores (such as the visual analogue scale, etc).
(2) Secondary outcome indicators: joint function scores (points-scoring systems such as Lysholm scores, Western Ontario and McMaster Universities Arthritis Index, etc), daily life quality scores (such as the MOS item short from health survey scores, etc), laboratory indicators (such as erythrocyte sedimentation rate, C-reactive protein, etc), adverse reaction.

### 2.5 Search strategy

**Figure 1:**
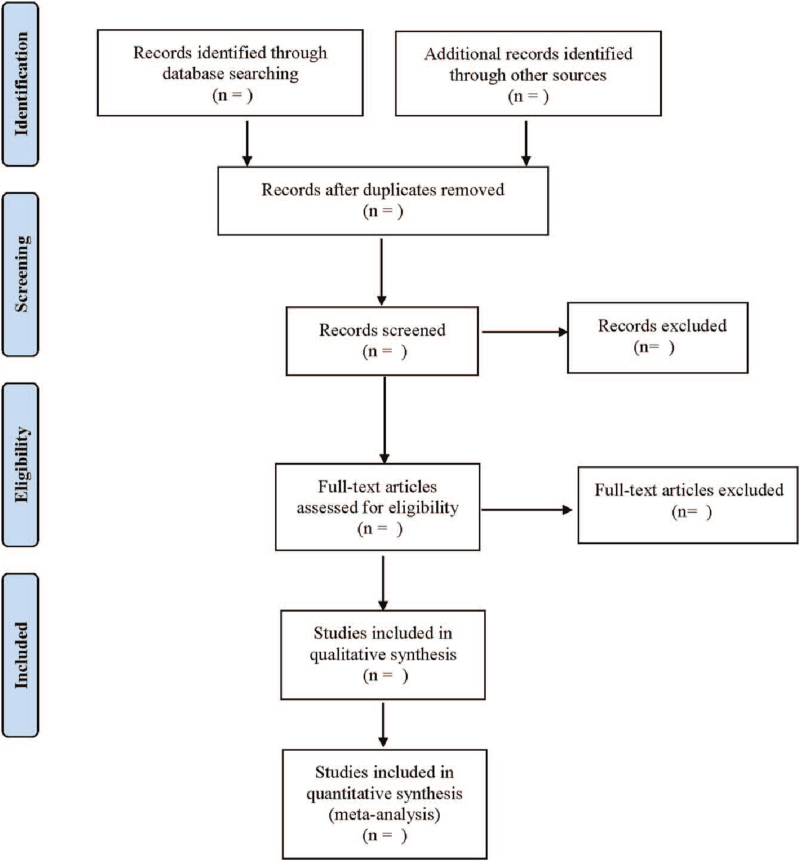
Flow diagram.

Two researchers independently searched for RCTs of acupuncture therapy in the treatment of RA from databases including PubMed, Embase, Web of Science, Cochrane Library, China National Knowledge Infrastructure, Wanfang database, from the establishment of the database to July 2021. Chinese search terms were “zhen ci”(acupuncture), “dian zhen”(electroacupuncture), “wen zhen jiu”(warm needle), “huo zhen”(fire needle), “ai jiu”(moxibustion), “er xue tie ya”(auricular application pressure), “er zhen”(auricular acupuncture), “xue wei mai xian”(acupoint catgut embedding), “xue wei zhu she”(acupoint injection), “lei feng shi xing guan jie yan”(rheumatoid arthritis). The English search term were “acupuncture,” “electroacupuncture,” “warm needle,” “fire needle,” “moxibustion,” “auricular application pressure,” “auricular needle,” “acupoint catgut embedding,” “acupoint injection,” “Rheumatoid arthritis,” “RA.” The included literature were independently screened by 2 researchers according to the inclusion and exclusion criteria. If there were differences of opinions, the decision would be made after consultation with the third researcher. The PubMed retrieval strategy is shown in Figure 1.

### 2.6 Data screening and extraction

Literature screening and data extraction were conducted independently and cross-checked by 2 researchers. Discuss and decide with a third researcher when there was a disagreement. The relevant information extracted was: first author, year of publication, diagnostic criteria for RA, sample size, gender, age, course of disease, research type, interventions, course of treatment, and outcome indicators. The literature screening process is shown in Table 1.

**Table 1:** Search strategy in PubMed database.

Number	Search terms
#1	Acupuncture [MeSH]
#2	Acupuncture [Title/Abstract]
#3	Pharmacopuncture [Title/Abstract]
#4	Electro-acupuncture [Title/Abstract]
#5	Warm needle [Title/Abstract]
#6	Fire needle [Title/Abstract]
#7	Blood-letting puncture [Title/Abstract]
#8	Moxibustion [MeSH]
#9	Moxibustion [Title/Abstract]
#10	Auricular application pressure [Title/Abstract]
#11	Auricular needle [Title/Abstract]
#12	Acupoint catgut embedding [Title/Abstract]
#13	Acupoint injection [Title/Abstract]
#14	Bee acupuncture [Title/Abstract]
#15	#1 OR #2 OR #3 OR #4 OR #5 OR #6 OR #7 OR #8 OR #9 OR #10 OR #11 OR #12 OR #13 OR #14
#16	Rheumatoid arthritis [MeSH]
#17	Rheumatoid Arthritis [Title/Abstract]
#18	RA [Title/Abstract]
#19	#16 OR #17 OR #18
#20	#15 AND #19

### 2.7 Literature quality assessment

The RCT bias risk assessment tool of Cochrane Manual of Systematic Reviews Version 5.1.0 was used to evaluate the quality of the included studies through random sequence generation, assignment concealment, blinding of patients, experimentalists, and outcome evaluators, the integrity of the resulting data, selective reporting, and other bias items. Two researchers evaluated the above contents as “low risk,” “high risk,” and “unclear,” cross-checked the evaluation results, and discussed with the third researcher if there were any differences, and no agreement could be reached. Finally, RevMan5.3 was used to draw the bias risk map.

### 2.8 Statistical analysis

Stata14.0 software was used to draw an evidence network map to show the comparison of the intervention measures for each outcome indicator. GeMTC14.3 based on the Bayesian framework was used for NMA. The effect values of dichonomical variables were represented by odds ratio, and the effective values of continuous variables were represented by mean difference. The 95% confidence interval was used to represent the statistical analysis results. A Markov Chain Monte Carlo fitting consistent model was used for Bayesian inference. Four chains were used for simulation, and the number of iterations was set as 50,000 (the first 20,000 for annealing and the last 30,000 for sampling). The potential scale reduction factor was used to reflect the convergence degree of the model. When the potential scale reduction factor was close to or equal to 1, it indicated that the data had good convergence and the obtained results were highly reliable.

### 2.9 Assessment of inconsistency

When there was a closed loop between the interventions, an inconsistency test was required. The *Z* test of Stata14.0 was used to evaluate the consistency of the results of direct comparison and indirect comparison. If *P* ≥ .05, it means that the possibility of inconsistency between direct comparison and indirect comparison is small. If *P* < .05, it means that there is a high possibility of inconsistency between direct comparison and indirect comparison, so fitting inconsistency analysis is needed. The surface under the cumulative ranking curves of different interventions was calculated by Stata14.0. The larger surface under the cumulative ranking curves value was, the better the efficacy of the intervention. Finally, a comparison-correction diagram should be drawn to evaluate the existence of a small sample effect.

### 2.10 Sensitivity analysis

Given that studies with different quality levels of the methodology may affect the final result, we conducted a sensitivity analysis by excluding studies with a high bias risk.

### 2.11 Assessment of publication bias

The comparison-adjusted funnel plots would be obtained with the specific ranking order to detect small sample size study effects and publication bias. All analyses would be conducted using R V.3.6.1 with the Gemtc package.

### 2.12 Evidence quality evaluation

We carried out the evidence grading of outcome indicators by means of Grading of Recommendation Assessment, Development, and Evaluation.^[17]^ The quality of evidence was rated on 4 levels: high, medium, low, or very low.

## 3 Discussion

At present, the pathogenesis of RA is not fully clear and may be related to autoimmune factors, infection, and genetics.^[18]^ No matter the length of the course of the disease, early effective treatment of RA patients can reduce the disability rate, control the patient's disease activities, help to prevent and delay the patient's condition, is also the key to the treatment of RA.^[19,20]^ Clinical observation found that in the process of use of non-steroidal anti-inflammatory drugs, anti-rheumatic drugs, and steroid hormone drugs, in addition to the toxic and side effects, they also show a gradual failure trend, and some patients will develop drug resistance, seriously reducing the therapeutic effect.^[21]^ Therefore, exploring safe and effective supplements and alternative therapy is urgent.

Acupuncture has always been regarded as an important part of traditional Chinese medicine, has been used for thousands of years to treat various clinical diseases, and has developed different acupuncture methods in clinical practice, and the application of different acupuncture methods has different effects. Acupuncture, for example, can effectively relieve pain and improve patients’ quality of life. Its efficacy is related to anti-inflammation, anti-oxidation, regulation of immune system function, and endorphins or serotonin^[11,22]^; warm needle can relieve the limb swelling of RA and effectively control the progression of the disease, which may be related to reducing the levels of serum immunoglobulin, IL-1, and TNF-α and alleviating the response^[23]^; fire needle can effectively control the inflammatory response of RA, with an effect similar to methotrexate, which is related to the down-regulation of ACPA and TNF-α levels.^[24]^ Since there is no direct comparison between different forms of acupuncture and moxibustion, differences in efficacy between different forms cannot be objectively evaluated, so it is inconvenient for the clinical selection of an appropriate acupuncture plan. Therefore, this study will explore the efficacy difference of RA through NMA and provide an evidence-based basis for clinical decision-makers to choose the optimal plan.

However, there are limitations in our study: due to the limitations of language retrieval, we included only the Chinese and English literature and may cause selection bias; staging, course of the disease, course of treatment of RA, and other factors may increase the possibility of heterogeneity. Nevertheless, we believe the results of this study will help find the best acupuncture regimen for RA.

### 3.1 Uncited Reference

^[25]^.

## Author contributions

**Conceptualization:** Jia Xu, Zilong Zhang.

**Data collection:** Jingwen Shang, Jia Xu.

**Funding acquisition:** Yongyang He.

**Funding support:** Yongyang He.

**Literature retrieval:** Jia Xu, Zilong Zhang.

**Software operating:** LinLing Tian, Yongyang He.

**Software:** LinLing Tian, Yongyang He.

**Supervision:** Jingwen Shang, Zilong Zhang.

**Writing – original draft:** Jingwen Shang, Jia Xu.

**Writing – review & editing:** Jingwen Shang, Yongyang He.
